# Sustainable intimate relationship beliefs in digital romantic media contexts: development and psychometric validation of the SIRBS among Chinese college students

**DOI:** 10.3389/fpsyg.2026.1929638

**Published:** 2026-10-28

**Authors:** Yanli Hua, Yihuan Yuan, Shengxuan Li, Dongming Li

**Affiliations:** 1 School of Languages and Culture, Hebei GEO University, Shijiazhuang, China; 2 College of Life Sciences, Hebei Normal University, Shijiazhuang, China

**Keywords:** Chinese college students, digital romantic media, media literacy, media psychology, psychometric validation, relationship beliefs, romantic scripts, sustainable intimate relationship beliefs

## Abstract

**Introduction:**

Digital romantic media may shape young adults' beliefs about intimacy, yet existing measures rarely assess relationship beliefs that jointly address autonomy, non-coercion, equality, and mutual growth. This study developed and validated the Sustainable Intimate Relationship Beliefs Scale (SIRBS) among Chinese college students.

**Methods:**

An online survey yielded a final analytic sample of 2,008 participants, which was randomly divided for exploratory and confirmatory factor analyses. Reliability, factor-level convergent and discriminant evidence, measurement invariance, criterion-related associations, mediation, and moderation were also examined.

**Results:**

The final 12-item scale comprised three correlated but conceptually distinct dimensions: Non-materialistic Relationship Beliefs, Low Control Tolerance, and Egalitarian Growth Beliefs. Confirmatory factor analysis supported the correlated three-factor model (CFI = 0.995, TLI = 0.993, RMSEA = 0.036, SRMR = 0.026). Internal consistency was satisfactory (Cronbach's alpha = 0.845; McDonald's omega total = 0.91), and composite reliability ranged from 0.878 to 0.967. Omega hierarchical (0.59) and explained common variance (0.39) indicated limited saturation by a single general factor. Approximate measurement invariance was supported across male-female gender groups and romantic relationship experience groups. SIRBS scores were positively associated with media literacy and negatively associated with unsustainable romantic-script internalization.

**Discussion:**

The findings support the three subscale scores as the primary basis for construct-specific interpretation. The total score may be used as a secondary broad composite but should not be interpreted as a strictly unidimensional trait.

## 1 Introduction

Young adulthood is a formative period for the development of romantic relationship beliefs, partner expectations, and assumptions about long-term intimacy (Arnett, 2000; Sprecher and Metts, 1989; Collins et al., 2009; Tartakovsky, 2023). These beliefs are psychologically important because they may shape how young adults evaluate potential partners, interpret relational boundaries, manage conflict, and imagine commitment (Fletcher et al., 1999; Knee, 1998; Yılmaz et al., 2023). For college students, relationship beliefs are still developing and may remain responsive to education, peer norms, personal experience, media exposure, and broader cultural change.

Digital media have become an increasingly important context in which young adults encounter models of romantic intimacy. Short videos, micro-dramas, social media platforms, and algorithmically recommended romantic content repeatedly present narratives about attraction, conflict, sacrifice, reconciliation, gender roles, and relational success (Bandura, 2001; Moyer-Gusé, 2008; Ward, 2003; Hefner and Wilson, 2013; Lippman et al., 2014). These narratives do not determine viewers' beliefs directly. However, they may make certain romantic scripts more cognitively and emotionally available, especially when viewers are immersed in narratives, identify with characters, or perceive a gap between idealized media romance and real or anticipated relationships (Cohen, 2001; Green and Brock, 2000).

Some romantic scripts in digital media may be psychologically problematic. Romantic content may idealize high-resource partners, frame material compensation as evidence of love, portray destiny as the basis of intimacy, romanticize possessiveness or emotional push-pull, or reproduce unequal gender agency (Moyer-Gusé, 2008; Ward, 2003; Hefner and Wilson, 2013; Lippman et al., 2014). When such scripts are internalized, young adults may be more likely to endorse relationship beliefs that are materialistic, control-tolerant, or gender-unequal. These beliefs are relevant not only to relationship education, but also to psychological wellbeing, autonomy, boundary recognition, and healthier relational functioning.

The present study uses the term sustainable intimate relationship beliefs to refer to psychological beliefs about romantic relationships that support dignity, autonomy, non-coercion, equality, mutual respect, and reciprocal development within intimate life (Pirrone et al., 2023; Gómez-López et al., 2019; Cubells-Serra et al., 2021). In this formulation, “sustainable” does not imply that individual beliefs directly determine macro-level sustainability outcomes. Rather, it denotes a psychologically and relationally sustainable orientation toward intimacy: one that resists materialized partner evaluation, rejects controlling relationship scripts, and endorses egalitarian mutual growth. This framing anchors relational sustainability in psychological needs, beliefs, and wellbeing while keeping the primary focus on media-related relationship scripts.

Existing measures capture several constructs adjacent to sustainable intimate relationship beliefs, including romantic beliefs, mate preferences, gender-role attitudes, relationship quality, marriage intention, materialism, acceptance of controlling behaviors, and media literacy (Fletcher et al., 1999; Richins and Dawson, 1992; Wolfe et al., 2001; Yılmaz et al., 2023). These instruments provide important assessments of particular attitudes, behaviors, or relationship outcomes, but they do not directly assess intimate relationship beliefs through an integrated framework combining non-materialism, low tolerance of control, and egalitarian mutual growth. This gap limits research on how young adults evaluate intimate relationships in ways that are psychologically healthy, socially equitable, and responsive to digital media environments.

The SIRBS is not intended to replace established measures of romantic idealization, general materialism, controlling behavior, or gender-role attitudes. Romantic-belief measures commonly emphasize idealization, destiny, and expectations about love; materialism measures assess a broader value orientation toward possessions and acquisition; control-related measures typically focus on the acceptance or experience of coercive behavior; and egalitarian-attitude measures address wider gender or social-role beliefs. In contrast, the SIRBS organizes relationship-specific beliefs from these adjacent domains into a brief multidimensional profile centered on partner evaluation, autonomy and control, and reciprocal development. Its distinctive contribution is therefore integrative and context-specific rather than a claim that the three domains are interchangeable manifestations of a single attitude.

To address this gap, the present study developed and validated the Sustainable Intimate Relationship Beliefs Scale (SIRBS) among Chinese college students. The SIRBS is conceptualized as a multidimensional measure of relationship beliefs comprising three theoretically related but distinguishable dimensions: Non-materialistic Relationship Beliefs, Low Control Tolerance, and Egalitarian Growth Beliefs.

Non-materialistic Relationship Beliefs refer to lower endorsement of material status, wealth, family background, consumption capacity, or appearance as primary criteria for evaluating partners or relationship quality. This dimension does not imply that economic stability is irrelevant to long-term relationships. Rather, it reflects resistance to reducing intimacy to material advantage or external status (Richins and Dawson, 1992; Dittmar et al., 2014; Tartakovsky, 2023). Such beliefs are psychologically relevant because excessive materialization of partner evaluation may intensify comparison, instrumental partner selection, and pressure around dating, love, and marriage.

Low Control Tolerance refers to rejection of possessive, coercive, surveillance-based, or autonomy-restricting behaviors in romantic relationships (Wolfe et al., 2001; Johnson, 1995; Stark, 2007; Pirrone et al., 2023). Some controlling behaviors may be misinterpreted or romanticized as care, devotion, protection, or passion. However, healthy intimate relationships require boundaries, trust, and respect for personal agency. Lower tolerance of control therefore reflects a belief orientation that values autonomy and mutual respect rather than dominance, dependence, or emotional coercion.

Egalitarian Growth Beliefs refer to endorsement of equality, reciprocal support, shared decision-making, communication, and mutual development within intimate relationships. This dimension captures a positive view of relationships as spaces in which both partners can maintain dignity, participate equally, and support each other's growth. It is closely related to gender equality and relational wellbeing, while remaining distinct from the rejection of materialism or control (Ridgeway and Correll, 2004; Glick and Fiske, 1996; Cubells-Serra et al., 2021).

These three dimensions are expected to be positively correlated because each reflects an orientation toward healthier and more sustainable intimacy. Nevertheless, they address different objects of evaluation: materialized partner standards, control and autonomy, and egalitarian mutual growth. The SIRBS was therefore specified primarily as a correlated multidimensional measure rather than as a strictly unidimensional scale. A correlated three-factor structure was expected to represent the instrument more appropriately than a single-factor structure, and the three subscales were designated as the principal scores for domain-specific interpretation.

**H1**. The SIRBS consists of three correlated dimensions: Non-materialistic Relationship Beliefs, Low Control Tolerance, and Egalitarian Growth Beliefs.

**H2**. The three-factor model of the SIRBS will fit the data better than a one-factor model.

As a newly developed measure, the SIRBS should demonstrate adequate psychometric quality (Boateng et al., 2018; DeVellis and Thorpe, 2022; Stefana et al., 2025). Specifically, the scale and its subdimensions should show acceptable internal consistency, convergent validity, and discriminant validity. Because the scale is intended for use among college students with different demographic and relationship backgrounds, it is also important to examine whether the measurement structure is comparable across key groups.

**H3**. The SIRBS will demonstrate adequate internal consistency, convergent validity, and discriminant validity.

**H4**. The SIRBS will demonstrate approximate measurement invariance across male–female gender groups and romantic relationship experience groups.

The nomological network of the SIRBS was examined in relation to digital romantic media-related experiences, unsustainable romantic-script internalization, media literacy, marriage intention, and marriage anxiety. Micro-drama exposure reflects the frequency or intensity of contact with romantic short-form media. Narrative transportation reflects cognitive and emotional immersion in romantic media narratives. Character involvement captures identification with or emotional attachment to romantic characters. Romantic reality gap refers to the perceived discrepancy between idealized media narratives and real or anticipated intimate relationships. These experiences may make media-based romantic scripts more salient and may be associated with weaker sustainable intimate relationship beliefs, especially when they are accompanied by stronger internalization of unsustainable scripts.

Media literacy provides a theoretically relevant positive correlate of sustainable intimate relationship beliefs. In the context of romantic digital media, media literacy refers to the ability to critically evaluate narrative exaggeration, commercial design, algorithmic recommendation, materialistic cues, controlling relationship scripts, and gendered representations (Livingstone, 2004; Jeong et al., 2012). Students with higher media literacy may be less likely to treat idealized or unequal romantic media narratives as realistic standards for intimate relationships. Accordingly, media literacy is expected to be positively associated with SIRBS scores and negatively associated with unsustainable romantic-script internalization.

**H5a**. The total SIRBS score will be positively associated with media literacy and negatively associated with unsustainable romantic-script internalization, micro-drama exposure, narrative transportation, character involvement, romantic reality gap, and marriage anxiety.

**H5b**. Because the three SIRBS dimensions capture theoretically distinct aspects of sustainable intimate relationship beliefs, the magnitude of these associations is expected to vary across subscales. In particular, media-engagement and romantic reality-gap variables are expected to show stronger associations with Non-materialistic Relationship Beliefs and Low Control Tolerance than with Egalitarian Growth Beliefs.

Beyond direct associations, unsustainable romantic-script internalization may help explain the relationship between digital media engagement and SIRBS scores (Bandura, 2001; Moyer-Gusé, 2008). Exposure to romantic media may not be problematic in itself; rather, the key issue may be whether viewers internalize the relationship scripts embedded in such content. Media literacy may also function as a protective resource (Jeong et al., 2012; Livingstone, 2004; Mallia et al., 2020). One possibility is that media literacy reduces the likelihood of internalizing unsustainable romantic scripts, thereby showing an indirect positive association with SIRBS scores. Another possibility is that media literacy weakens the negative association between already-internalized unsustainable scripts and SIRBS scores. Because these mechanisms are theoretically plausible but empirically uncertain, the mediating role of script internalization and the moderating role of media literacy are examined as research questions.

**RQ1**. Does unsustainable romantic-script internalization mediate the associations of digital romantic media-related experiences and media literacy with SIRBS scores?

**RQ2**. Does media literacy moderate the association between unsustainable romantic-script internalization and SIRBS scores?

Using a large sample and a split-half validation design, this study evaluated the factor structure, reliability, validity, measurement invariance, and nomological associations of the SIRBS. By providing a brief multidimensional measure, this study aims to advance psychological research on intimate relationship beliefs, digital romantic media, media literacy, and relationship education among college students.

Together, these hypotheses and research questions are summarized in Figure 1.

**Figure 1:**
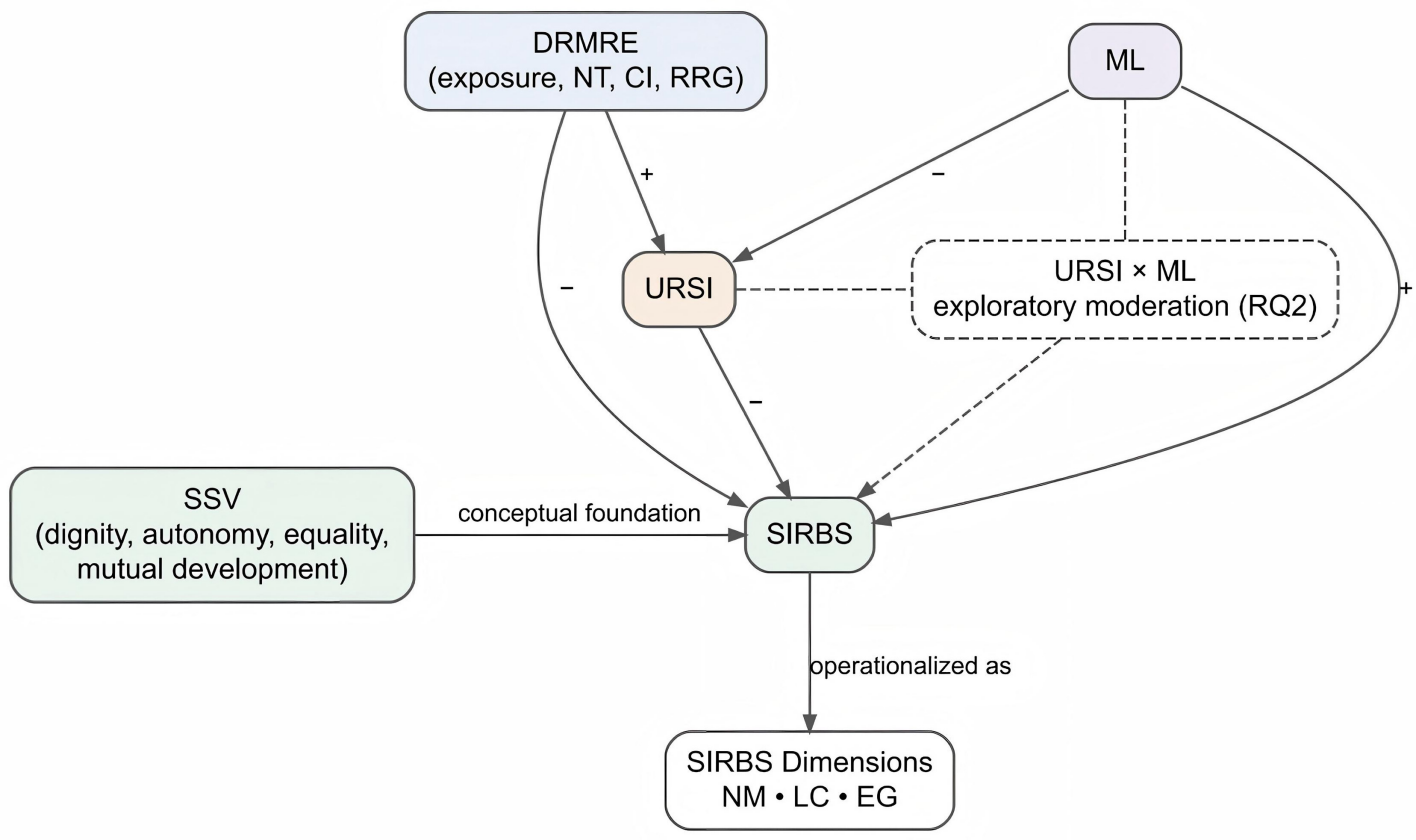
Theoretical and nomological framework of the Sustainable Intimate Relationship Beliefs Scale. SSV, social sustainability values; DRMRE, digital romantic media-related experiences; NT, narrative transportation; CI, character involvement; RRG, romantic reality gap; URSI, unsustainable romantic-script internalization; ML, media literacy; SIRBS, Sustainable Intimate Relationship Beliefs Scale; NM, Non-materialistic Relationship Beliefs; LC, Low Control Tolerance; EG, Egalitarian Growth Beliefs. Solid arrows represent theoretically expected associations. Dashed arrows represent the exploratory moderation pathway. Plus and minus signs indicate the hypothesized direction of association.

## 2 Materials and methods

### 2.1 Participants and procedure

Data were collected from 8 to 15 May 2026 using an anonymous online questionnaire distributed through a WeChat-linked survey channel. Eligible participants were at least 18 years old and enrolled in a higher education institution. Of the 2,152 submitted responses, 2,008 were retained after applying the eligibility, completion-time, attention-check, and SIRBS-completeness criteria. One response was excluded because the participant was younger than 18 years. All remaining submitted cases met the student-status eligibility criterion and had complete responses on the 12 SIRBS items. Sixty-four responses were excluded because completion time was shorter than the pre-specified 120-s threshold, and 79 additional responses were excluded because they did not provide the instructed response to the attention-check item. The full data-screening procedure and completion-time screening summary are reported in Supplementary Tables S3c, d.

The study was reviewed and approved by the School of Languages and Culture, Hebei GEO University, on 22 April 2025. The approval was granted for an anonymous online questionnaire study involving students aged 18 years or older who were enrolled in higher education institutions. The study involved no clinical intervention, medical diagnosis or treatment, collection of biological samples, or testing of drugs or medical devices, and the level of risk was judged to be no greater than that ordinarily encountered in daily life. No formal approval code was issued. Before accessing the questionnaire, all participants reviewed an information sheet and provided electronic informed consent by selecting “I agree to participate.” Participation was voluntary, and participants could withdraw at any time without adverse consequences. No compensation was provided.

For psychometric validation, the analytic sample was randomly divided into two equal subsamples using a fixed random seed. Study 1 was used for exploratory factor analysis and initial item evaluation; Study 2 was used for confirmatory factor analysis and model comparison.

### 2.2 Item generation and scoring

The SIRBS was designed to assess sustainable intimate relationship beliefs, defined as endorsement of non-materialistic, low-control, egalitarian, mutually respectful, and growth-oriented beliefs about romantic relationships. Candidate items were drafted to represent four conceptual domains identified from the scale-development and substantive literature (Boateng et al., 2018; DeVellis and Thorpe, 2022; Stefana et al., 2025): relational wellbeing, autonomy and boundary recognition, gender equality and egalitarian partnership, and critical media literacy regarding romantic media. Item wording was tailored to belief statements that Chinese college students could evaluate without requiring current relationship experience.

The initial item pool was reviewed by two experts in educational psychology (Boateng et al., 2018; Clark and Watson, 1995; Stefana et al., 2025). The experts evaluated each item for conceptual relevance, clarity, cultural appropriateness, redundancy, suitability for Chinese college students, and alignment with its intended domain. Items were revised or removed when they overlapped conceptually with another item, used ambiguous or potentially leading wording, or did not represent the intended belief domain clearly. A cognitive debriefing with 28 college students then examined comprehensibility, interpretation, and the suitability of the response format. Feedback was used to refine wording where necessary and to confirm that the items were interpreted consistently with their intended meaning. This process yielded a balanced 12-item form with four items representing each of the three dimensions. The item-development and content-review process is summarized in Supplementary Table S2a.

The final scale contains three four-item dimensions: Non-materialistic Relationship Beliefs (NM), Low Control Tolerance (LC), and Egalitarian Growth Beliefs (EG). Items are rated from 1 = strongly disagree to 5 = strongly agree. NM and LC items are reverse-scored, whereas EG items are forward-scored. Each subscale score is the mean of its four items and constitutes the primary score for interpreting the corresponding belief domain. A total SIRBS score can be calculated as the mean of all 12 scored items when a broad descriptive composite is useful; however, it should be reported alongside the subscale scores and should not be interpreted as a strictly unidimensional latent trait. Higher scores indicate more sustainable intimate relationship beliefs at the relevant level of interpretation. Full Chinese items and English translations are provided in Supplementary Table S1.

### 2.3 External validation measures

All external validation variables were assessed using self-report items rated on a five-point Likert-type scale unless otherwise noted. Mean scores were computed so that higher values indicated higher levels of the target construct.

External variables were included to provide an initial nomological and criterion-related evaluation of the SIRBS. They comprised micro-drama exposure, narrative transportation, character involvement, romantic reality gap, unsustainable romantic-script internalization, media literacy, marriage intention, and marriage anxiety. Several measures were developed for the present research context and were therefore used to test theoretically expected patterns of association rather than to establish convergence with mature external instruments. Item domains, scoring procedures, and internal consistency coefficients are reported in Supplementary Table S2.

### 2.4 Data analysis

Analyses were conducted in R using the psych, lavaan, semTools, dplyr, and openxlsx packages. Item descriptives included means, standard deviations, skewness, kurtosis, and floor/ceiling effects; floor or ceiling proportions above 15% were treated as potentially problematic.

In Study 1, exploratory factor analysis was performed using principal axis factoring with oblimin rotation (Fabrigar et al., 1999). Factorability was evaluated with the Kaiser-Meyer-Olkin statistic and Bartlett's test of sphericity, and factor retention was guided by parallel analysis. Items were evaluated using primary loadings, cross-loadings, and theoretical alignment.

In Study 2, confirmatory factor analysis was conducted using WLSMV estimation for ordered Likert-type indicators. A one-factor model, correlated three-factor model, second-order model, and bifactor model were compared using chi-square, CFI, TLI, RMSEA, and SRMR (Brown, 2015; Flora and Curran, 2004; Hu and Bentler, 1999). CFI/TLI values above 0.90 and 0.95 were treated as acceptable and good fit, respectively; RMSEA/SRMR values below 0.08 and 0.06 were treated as acceptable and good fit, respectively.

Internal consistency was evaluated using Cronbach's alpha and McDonald's omega (Fornell and Larcker, 1981; Henseler et al., 2015; McNeish, 2018). Dimensionality was further examined using omega hierarchical and explained common variance. Composite reliability and average variance extracted were used to evaluate factor-level convergence within the measurement model; the Fornell-Larcker criterion and heterotrait-monotrait ratios were used to evaluate discrimination among the three latent dimensions. Associations with external variables were interpreted separately as preliminary nomological and criterion-related evidence.

Measurement invariance was tested across gender and romantic relationship experience (Chen, 2007; Cheung and Rensvold, 2002). Because sparse extreme categories produced subgroup estimation instability, invariance models used MLR with items treated as approximately continuous. Therefore, the invariance findings were interpreted as evidence for approximate configural, metric, and scalar invariance rather than strict ordinal threshold invariance. Configural, metric, and scalar models were compared using changes in CFI, RMSEA, and SRMR.

Criterion-related validity was examined using Pearson correlations. Incremental validity was tested with hierarchical regressions predicting marriage intention and marriage anxiety after demographic covariates and media/script variables were entered. Mediation models tested indirect associations through unsustainable romantic-script internalization using maximum likelihood estimation with 5,000 bootstrap samples. Exploratory moderation was tested by adding the interaction between unsustainable romantic-script internalization and media literacy. Common method bias was examined with Harman's single-factor test and a single-factor CFA diagnostic model (Podsakoff et al., 2003). Robustness was assessed through 50 random split-half CFA replications.

## 3 Results

### 3.1 Sample characteristics

The analytic sample included 2008 Chinese college students (Table 1). The EFA and CFA subsamples each included 1,004 participants.

**Table 1:** Sample characteristics.

Characteristic	Category	n/M	%/SD
Total sample	Final analytic sample	2,008	100.0
Split-half sample	Study 1: EFA sample	1,004	50.0
Split-half sample	Study 2: CFA sample	1,004	50.0
Gender	Male	857	42.7
Gender	Female	1,150	57.3
Gender	Other	1	0.1
Age	Mean age	20.43	1.87
Age	Range	18–28	—
Romantic relationship experience	Yes	1,594	79.4
Romantic relationship experience	No	414	20.6

*N* = 2,008. Percentages are based on the final analytic sample and may not sum to 100 because of rounding. M, mean; SD, standard deviation. Study 1 and Study 2 were generated using a random split-half procedure with approximately equal subsample sizes.

### 3.2 Item descriptives and reliability

Item descriptive statistics are reported in Table 2. Item means ranged from 3.054 to 3.219, standard deviations from 0.589 to 0.641, skewness from −0.050 to 0.109, and kurtosis from −0.053 to 0.261. Floor and ceiling effects were below 15% for all items. Corrected item-total correlations ranged from 0.612 to 0.816, and alpha-if-item-deleted values did not exceed the corresponding subscale alpha values (Supplementary Table S3a).

**Table 2:** Item descriptive statistics for the SIRBS items (*N* = 2,008).

Item	M	SD	Skewness	Kurtosis
NM1	3.102	0.612	0.045	−0.026
NM2	3.097	0.601	0.013	0.126
NM3	3.105	0.612	0.056	−0.001
NM4	3.102	0.619	−0.044	−0.053
LC1	3.196	0.611	0.010	0.134
LC2	3.204	0.607	0.028	0.090
LC3	3.219	0.589	0.109	−0.038
LC4	3.214	0.591	0.048	0.147
EG1	3.055	0.622	−0.037	0.051
EG2	3.073	0.625	−0.028	−0.002
EG3	3.080	0.641	−0.050	0.107
EG4	3.054	0.613	0.008	0.261

*N* = 2,008. M, mean; SD, standard deviation; NM, Non-materialistic Relationship Beliefs; LC, Low Control Tolerance; EG, Egalitarian Growth Beliefs. All items were rated on a five-point Likert-type scale. NM and LC items were reverse-scored before analysis, so higher item values indicate stronger sustainable intimate relationship beliefs.

Detailed response-category distributions and distribution diagnostics for the 12 SIRBS items are provided in Supplementary Tables S3b-1, b-2, respectively. Although floor and ceiling effects were low, responses were centrally concentrated around the midpoint category, which should be considered when interpreting the item distributions. Pearson and polychoric inter-item correlation matrices are reported in Supplementary Tables S3f, g. Reliability estimates for the final analytic sample after eligibility and quality-control exclusions are reported in Supplementary Table S3e.

Reliability coefficients are shown in Table 3. Cronbach's alpha ranged from 0.806 to 0.909 across subscales, and McDonald's omega ranged from 0.81 to 0.91. For the total SIRBS composite, alpha was 0.845 and omega total was 0.91. Omega hierarchical was 0.59, and explained common variance was 0.39. Thus, the composite showed satisfactory overall-score reliability, but only a moderate proportion of reliable variance was attributable to a single general factor.

**Table 3:** Reliability coefficients by SIRBS dimension.

Dimension	Number of items	Cronbach's α	McDonald's ω
NM	4	0.909	0.91
LC	4	0.806	0.81
EG	4	0.809	0.81
Total SIRBS	12	0.845	0.91

NM, Non-materialistic Relationship Beliefs; LC, Low Control Tolerance; EG, Egalitarian Growth Beliefs; SIRBS, Sustainable Intimate Relationship Beliefs Scale. Reliability coefficients were computed using the scored items. The omega value for the total SIRBS refers to McDonald's omega total.

### 3.3 Exploratory factor analysis

In Study 1, the KMO value was 0.876, and Bartlett's test of sphericity was significant, χ^2^_(66)_ = 5638.542, *p* < 0.001. Parallel analysis indicated a three-factor solution. Primary factor loadings exceeded 0.66, and absolute cross-loadings were below 0.05. The three factors corresponded to NM, LC, and EG (Supplementary Table S4).

### 3.4 Confirmatory factor analysis and model comparison

CFA results for Study 2 are reported in Table 4. The one-factor model showed poor fit, whereas the correlated three-factor, second-order, and bifactor models showed good global fit.

**Table 4:** CFA model fit comparison in Study 2.

Model	χ^2^	df	CFI	TLI	RMSEA	SRMR
Single-factor model	1648.32	54	0.884	0.858	0.171	0.129
Three-factor correlated model	118.46	51	0.995	0.993	0.036	0.026
Second-order model	118.46	51	0.995	0.993	0.036	0.026
Bifactor model	68.71	42	0.998	0.996	0.025	0.017

Models were estimated in the CFA subsample. CFI, comparative fit index; TLI, Tucker–Lewis index; RMSEA, root mean square error of approximation; SRMR, standardized root mean square residual. The second-order model with three first-order factors produced fit indices identical to those of the correlated three-factor model under this specification. The second-order model was not interpreted as separate evidence for a higher-order structure because, with only three first-order factors, it was empirically equivalent to the correlated three-factor model under this specification.

Although the bifactor model showed slightly better global fit, omega hierarchical (0.59) and explained common variance (0.39) provided limited support for a predominantly unidimensional interpretation. The modest correlations among the three dimensions, their theoretical distinctiveness, and the greater parsimony of the correlated three-factor solution further favored a multidimensional scoring interpretation. The correlated three-factor model was therefore retained as the primary measurement model. Subscale scores were treated as the principal scores for substantive interpretation, whereas the total score was retained only as a secondary broad composite.

Standardized factor loadings, composite reliability, and average variance extracted are reported in Table 5. Standardized loadings ranged from 0.768 to 0.955. Composite reliability ranged from 0.878 to 0.967, and AVE ranged from 0.643 to 0.880.

**Table 5:** Standardized CFA loadings, composite reliability, and average variance extracted.

Item	Standardized loading	CR	AVE
NM1	0.926	0.967	0.88
NM2	0.917		
NM3	0.954		
NM4	0.955		
LC1	0.790	0.887	0.662
LC2	0.852		
LC3	0.792		
LC4	0.820		
EG1	0.768	0.878	0.643
EG2	0.801		
EG3	0.840		
EG4	0.797		

NM, Non-materialistic Relationship Beliefs; LC, Low Control Tolerance; EG, Egalitarian Growth Beliefs; CR, composite reliability; AVE, average variance extracted. Standardized loadings are reported for the retained three-factor model. CR and AVE are reported once for each latent dimension; blank cells indicate values belonging to the same dimension.

Latent correlations among the three dimensions ranged from 0.236 to 0.396. The square root of AVE for each dimension exceeded its correlations with other dimensions. HTMT ratios ranged from 0.275 to 0.462 (Supplementary Table S5).

### 3.5 Measurement invariance

Measurement invariance results are shown in Table 6. Across gender, configural, metric, and scalar models showed acceptable fit; ΔCFI ranged from −0.001 to 0.001, ΔRMSEA from −0.004 to 0.001, and ΔSRMR from 0.001 to 0.003. Across romantic relationship experience, ΔCFI was 0.000, ΔRMSEA ranged from −0.002 to 0.003, and ΔSRMR ranged from 0.000 to 0.004. Observed group comparisons after scalar invariance are reported in Supplementary Table S6.

**Table 6:** Approximate measurement invariance across male–female gender groups and romantic relationship experience groups.

Grouping variable	Model	CFI	TLI	RMSEA	SRMR	ΔCFI	ΔRMSEA	ΔSRMR
Gender	Configural	1	0.999	0.007	0.016	–	–	–
Gender	Metric	0.999	0.999	0.008	0.019	−0.001	0.001	0.003
Gender	Scalar	1	1	0.004	0.02	0.001	−0.004	0.001
Relationship experience	Configural	0.999	0.999	0.007	0.015	–	–	–
Relationship experience	Metric	0.999	0.999	0.01	0.019	0	0.003	0.004
Relationship experience	Scalar	0.999	0.999	0.008	0.019	0	−0.002	0

CFI, comparative fit index; TLI, Tucker–Lewis index; RMSEA, root mean square error of approximation; SRMR, standardized root mean square residual. Gender invariance models included male and female participants; the participant identifying as other was not included because of the very small cell size. Relationship experience groups included participants with and without prior or current romantic relationship experience. Δ values indicate changes from the preceding invariance model.

### 3.6 Criterion-related and incremental validity

Pearson correlations are reported in Table 7. The total SIRBS score correlated positively with media literacy (*r* = 0.504) and marriage intention (*r* = 0.112), and negatively with unsustainable romantic-script internalization (*r* = −0.618), romantic reality gap (*r* = −0.476), character involvement (*r* = −0.402), narrative transportation (*r* = −0.394), micro-drama exposure (*r* = −0.295), and marriage anxiety (*r* = −0.288).

**Table 7:** Pearson correlations between SIRBS scores and external variables.

External variable	Total SIRBS	NM	LC	EG
Micro-drama exposure	−0.295	−0.329	−0.262	−0.053
Narrative transportation	−0.394	−0.501	−0.286	−0.065
Character involvement	−0.402	−0.452	−0.331	−0.093
Romantic reality gap	−0.476	−0.571	−0.364	−0.097
Unsustainable romantic-script internalization	−0.618	−0.478	−0.477	−0.410
Media literacy	0.504	0.441	0.341	0.324
Marriage intention	0.112	−0.035	0.067	0.225
Marriage anxiety	−0.288	−0.299	−0.233	−0.097

*N* = 2,008. Values are Pearson correlation coefficients. SIRBS, Sustainable Intimate Relationship Beliefs Scale; NM, Non-materialistic Relationship Beliefs; LC, Low Control Tolerance; EG, Egalitarian Growth Beliefs. NM and LC items were reverse-scored before computing subscale and total scores, so higher scores indicate less materialistic partner evaluation and lower tolerance of controlling relationship scripts. Higher EG scores indicate stronger endorsement of egalitarian growth beliefs.

At the subscale level, NM showed the strongest negative correlations with romantic reality gap (*r* = −0.571), narrative transportation (*r* = −0.501), and character involvement (*r* = −0.452). LC correlated negatively with unsustainable romantic-script internalization (*r* = −0.477), romantic reality gap (*r* = −0.364), and character involvement (*r* = −0.331). EG showed its strongest positive correlation with marriage intention (*r* = 0.225), while its correlations with media-engagement variables were relatively small.

In hierarchical regression models, adding the total SIRBS score explained an additional 2.8% of the variance in marriage intention, Δ*R*^2^ = 0.028, *p* < 0.001. The incremental contribution to marriage anxiety was Δ*R*^2^ = 0.002, *p* =0.055. Thus, incremental validity evidence was mixed: the SIRBS showed a significant incremental contribution to marriage intention, whereas its additional contribution to marriage anxiety was small and did not reach conventional significance. Full regression results are reported in Supplementary Tables S7a, b.

Overall, H5a was supported for the total SIRBS composite. The composite was positively associated with media literacy and negatively associated with unsustainable romantic-script internalization, romantic reality gap, character involvement, narrative transportation, micro-drama exposure, and marriage anxiety. However, the pattern and magnitude of associations differed across the three subscales, consistent with H5b. Associations with media-engagement and romantic reality-gap variables were strongest for Non-materialistic Relationship Beliefs, moderate for Low Control Tolerance, and weakest for Egalitarian Growth Beliefs. These differences reinforce the primacy of the subscale scores and show that the three dimensions should not be treated as interchangeable, even when a broad composite is reported descriptively.

### 3.7 Mediation analysis

Direct and indirect effects are reported in Table 8. Unsustainable romantic-script internalization was negatively associated with SIRBS scores (B = −0.411, *p* < 0.001). Media literacy was positively associated with SIRBS scores (B = 0.259, *p* < 0.001) and negatively associated with unsustainable romantic-script internalization (B = −0.281, *p* < 0.001). Micro-drama exposure, character involvement, and romantic reality gap were positively associated with unsustainable romantic-script internalization; narrative transportation was not.

**Table 8:** Direct and indirect effects in the mediation model.

Panel A. Direct effects	B	SE	*p*
Unsustainable romantic-script internalization ← Micro-drama exposure	0.082	0.022	< 0.001
Unsustainable romantic-script internalization ← Narrative transportation	0.008	0.026	0.748
Unsustainable romantic-script internalization ← Character involvement	0.171	0.024	< 0.001
Unsustainable romantic-script internalization ← Romantic reality gap	0.153	0.028	< 0.001
Unsustainable romantic-script internalization ← Media literacy	−0.281	0.02	< 0.001
SIRBS ← Unsustainable romantic-script internalization	−0.411	0.017	< 0.001
SIRBS ← Micro-drama exposure	−0.031	0.018	0.075
SIRBS ← Narrative transportation	−0.089	0.021	< 0.001
SIRBS ← Character involvement	−0.061	0.02	0.002
SIRBS ← Romantic reality gap	−0.135	0.022	< 0.001
SIRBS ← Media literacy	0.259	0.018	< 0.001
Panel B. Indirect effects	B	95% bootstrap CI	p
Micro-drama exposure → Unsustainable romantic-script internalization → SIRBS	−0.034	[−0.053, −0.016]	< 0.001
Narrative transportation → Unsustainable romantic-script internalization → SIRBS	−0.003	[−0.025,0.017]	0.748
Character involvement → Unsustainable romantic-script internalization → SIRBS	−0.070	[−0.091, −0.051]	< 0.001
Romantic reality gap → Unsustainable romantic-script internalization → SIRBS	−0.063	[−0.087, −0.040]	< 0.001
Media literacy → Unsustainable romantic-script internalization → SIRBS	0.115	[0.096, 0.134]	< 0.001

SIRBS, Sustainable Intimate Relationship Beliefs Scale; B, unstandardized coefficient; SE, standard error; CI, confidence interval. Continuous predictors were standardized before model estimation. Indirect effects were tested using 5,000 bootstrap samples, and 95% bootstrap confidence intervals are reported.

Significant indirect effects through unsustainable romantic-script internalization were observed for micro-drama exposure (B = −0.034, 95% CI [−0.053, −0.016]), character involvement (B = −0.070, 95% CI [−0.091, −0.051]), romantic reality gap (B = −0.063, 95% CI [−0.087, −0.040]), and media literacy (B = 0.115, 95% CI [0.096, 0.134]). The indirect effect of narrative transportation was not significant (B = −0.003, 95% CI [−0.025, 0.017]).

Thus, RQ1 was partially supported: unsustainable romantic-script internalization significantly mediated the associations of micro-drama exposure, character involvement, romantic reality gap, and media literacy with SIRBS scores, but it did not mediate the association between narrative transportation and SIRBS scores.

### 3.8 Exploratory moderation analysis

The interaction between unsustainable romantic-script internalization and media literacy was not significant (B = 0.013, *p* = 0.366; Supplementary Table S8). In the same model, unsustainable romantic-script internalization was negatively associated with SIRBS scores, and media literacy was positively associated with SIRBS scores. Therefore, RQ2 was not supported. Media literacy did not significantly moderate the association between unsustainable romantic-script internalization and SIRBS scores.

### 3.9 Common method bias and robustness checks

Harman's single-factor test showed that the first unrotated factor explained 21.9% of the variance. The single-factor CFA diagnostic model showed poor fit: CFI = 0.457, TLI = 0.438, RMSEA = 0.095, and SRMR = 0.105 (Supplementary Table S9a).

Across 50 random split-half CFA replications, the mean fit indices for the three-factor model were CFI = 0.992 (SD = 0.004), TLI = 0.989 (SD = 0.006), RMSEA = 0.034 (SD = 0.008), and SRMR = 0.028 (SD = 0.004; Supplementary Table S9b).

## 4 Discussion

### 4.1 Principal findings

This study developed and validated the Sustainable Intimate Relationship Beliefs Scale (SIRBS), a brief multidimensional instrument for assessing young adults' psychologically and relationally sustainable beliefs about intimate relationships. The psychometric evidence supported a 12-item, correlated three-factor structure comprising Non-materialistic Relationship Beliefs, Low Control Tolerance, and Egalitarian Growth Beliefs. The scale showed strong factorial validity, satisfactory internal consistency, factor-level convergent and discriminant evidence, and approximate measurement invariance across male–female gender groups and romantic relationship experience groups. Evidence for the total score was more qualified: although omega total was high, omega hierarchical and explained common variance did not support interpreting the instrument as predominantly unidimensional.

Beyond establishing a measurement model, the findings locate the SIRBS within a theoretically meaningful network of digital media and relationship-related variables. Higher SIRBS scores were associated with stronger media literacy and lower levels of micro-drama exposure, unsustainable romantic-script internalization, romantic reality gap, narrative transportation, character involvement, and marriage anxiety. The mediation results provided partial support for the proposed script-internalization pathway: unsustainable romantic-script internalization accounted for the associations of micro-drama exposure, character involvement, romantic reality gap, and media literacy with SIRBS scores, but not the association involving narrative transportation. Taken together, these findings suggest that the SIRBS captures a coherent but multidimensional belief system relevant to the formation of healthier and more sustainable relational cultures.

### 4.2 Contribution to psychological research on intimate relationship beliefs

A central contribution of this study is to conceptualize sustainable intimate relationship beliefs as a multidimensional psychological construct. Rather than treating sustainability as a macro-level outcome, the SIRBS operationalizes a set of relationship beliefs that are relevant to autonomy, boundary recognition, equality, non-coercion, and mutual development in intimate life. This framing connects relationship beliefs with psychological research on romantic expectations, gendered relational norms, media-influenced scripts, and youth wellbeing.

The three SIRBS dimensions translate these psychological concerns into measurable domains: resistance to materialized partner evaluation, low tolerance of controlling relationship scripts, and endorsement of egalitarian mutual growth. The dimensions were related but not interchangeable, which supports a profile-based interpretation centered on the three subscales. A total score can summarize the broad direction of these beliefs, but it necessarily combines distinguishable domains and should not replace dimension-specific interpretation in research or practice.

### 4.3 Psychometric and measurement implications

The findings have several measurement implications (Boateng et al., 2018; DeVellis and Thorpe, 2022; Stefana et al., 2025). By assessing non-materialistic relationship beliefs, low control tolerance, and egalitarian growth beliefs within one concise instrument, the SIRBS enables researchers to examine sustainable intimacy as a multidimensional construct rather than as a general attitude toward romantic relationships. The correlated factor structure and differentiated external associations support the use of the three subscale scores as the primary basis for substantive interpretation.

The total SIRBS score may be used as a secondary broad descriptive composite when the research question concerns the overall direction of sustainable relationship beliefs. Its interpretation should nevertheless remain explicitly multidimensional. Omega hierarchical and explained common variance did not support treating the total score as a strongly unidimensional trait, and different students may obtain similar totals while showing different profiles across NM, LC, and EG. Researchers should therefore report the three subscale scores whenever construct-specific conclusions are drawn and, when a total score is reported, present it alongside the subscales rather than as a substitute for them.

The evidence for approximate measurement invariance across male–female gender groups and romantic relationship experience further strengthens the utility of the scale (Chen, 2007; Cheung and Rensvold, 2002). Invariance indicates that the SIRBS can be used to compare these groups with greater confidence and to examine whether the associations between sustainable relationship beliefs and external variables differ across groups. This is particularly useful for sustainability-oriented education and intervention research, where researchers may need to evaluate whether programs operate similarly across the gender and relationship-experience groups examined in this study.

### 4.4 Digital romantic scripts and sustainable relationship beliefs

The findings also clarify how digital romantic media can be incorporated into the study of sustainable intimate relationship beliefs. The results do not support a simple exposure-based interpretation in which contact with romantic micro-dramas or short-form romantic content automatically weakens sustainable relationship beliefs. Rather, the more theoretically relevant process appears to involve the internalization of unsustainable romantic scripts. Media-related experiences were associated with lower SIRBS scores particularly when they were linked to stronger endorsement of scripts emphasizing materialized partner standards, dramatic conflict compensation, possessive control, or unequal agency.

For theory and application, the key implication is that media exposure alone is less informative than the internalization of specific romantic scripts. Digital media are not merely channels of entertainment; they are cultural environments in which young adults repeatedly encounter models of attraction, conflict, commitment, gender relations, and relational success (Bandura, 2001; Ward, 2003; Hefner and Wilson, 2013; Lippman et al., 2014). Some of these models may support autonomy, respect, and equality, whereas others may normalize control, consumption, or asymmetric sacrifice as signs of love. By showing that unsustainable script internalization was strongly and negatively associated with SIRBS scores, this study highlights a cultural-psychological mechanism through which digital romantic narratives may become linked to young adults' relationship beliefs and expectations.

The pattern for narrative transportation deserves particular attention (Moyer-Gusé, 2008; Green and Brock, 2000). Transportation was directly associated with lower SIRBS scores, but its indirect effect through unsustainable script internalization was not significant. This suggests that immersive engagement with romantic narratives may operate through processes not fully captured by the internalization measure, such as emotional idealization, upward comparison, fantasy involvement, or temporary recalibration of relationship expectations. Future studies should distinguish different forms of media engagement and examine whether reflective, critical, or socially contextualized engagement has different implications from uncritical immersion.

### 4.5 Media literacy as an upstream protective resource

Media literacy emerged as a key protective correlate in the nomological network of the SIRBS (Livingstone, 2004; Jeong et al., 2012). Students with higher media literacy reported stronger sustainable intimate relationship beliefs and lower unsustainable romantic-script internalization. The indirect association between media literacy and SIRBS through lower script internalization suggests that media literacy may help students resist the normalization of materialistic, controlling, or gender-unequal romantic scripts. This finding is consistent with the view that critical interpretation of media content can support healthier relationship expectations in digitally saturated environments.

The non-significant moderation result suggests that media literacy may operate earlier in the process, by reducing the likelihood that students internalize materialistic, controlling, or gender-unequal romantic scripts (Jeong et al., 2012; Livingstone, 2004; Mallia et al., 2020). Educational programs may therefore be more effective when they combine relationship education with critical analysis of digital romantic narratives, rather than relying only on warnings about media exposure.

### 4.6 Relationship education and student wellbeing

The SIRBS offers a concise instrument for assessing psychologically healthier relationship beliefs in higher education settings. The scale may inform relationship education, student wellbeing programs, counseling-oriented screening, and gender equality initiatives (Gómez-López et al., 2019; Cubells-Serra et al., 2021). Because the SIRBS distinguishes among non-materialistic relationship beliefs, low tolerance of controlling relationship scripts, and egalitarian growth beliefs, it can help educators and practitioners identify specific belief domains that may require support. Lower NM scores may indicate stronger materialized partner-evaluation standards, lower LC scores may suggest greater acceptance of possessive or coercive scripts, and lower EG scores may point to weaker endorsement of mutual respect, communication, and shared decision-making.

As a 12-item multidimensional measure, the SIRBS is also suitable for large-scale surveys, needs assessment, and program evaluation. Its brevity reduces respondent burden while preserving information on three theoretically distinct domains of sustainable intimate relationship beliefs. Because approximate measurement invariance was supported across male–female gender groups and romantic relationship experience groups, the subscale scores may be used to compare the groups examined in this study or to evaluate whether educational programs benefit them in similar ways. The total composite may supplement, but should not replace, these domain-level comparisons.

### 4.7 Media literacy and intervention evaluation

The SIRBS may also be used to evaluate media literacy interventions focused on digital romantic narratives (Jeong et al., 2012; Mallia et al., 2020). Because the findings suggest that unsustainable script internalization is more relevant than exposure alone, the scale can help assess whether students become better able to distinguish entertainment conventions from psychologically healthier relationship beliefs grounded in autonomy, equality, mutual respect, and long-term relational wellbeing.

In practice, the SIRBS can serve as a pre- and post-intervention outcome measure (Mallia et al., 2020) in programs that teach students to critically analyze how short videos, micro-dramas, and algorithmically recommended romantic content construct desirability, conflict, sacrifice, control, and gendered agency.

### 4.8 Limitations and future research

Several limitations should be considered when interpreting the findings. First, the cross-sectional design limits causal inference (Maxwell and Cole, 2007). Although the mediation results were theoretically specified and consistent with a script-internalization pathway, the temporal ordering among digital media engagement, media literacy, script internalization, and sustainable intimate relationship beliefs cannot be established. Longitudinal and experimental studies are needed to examine whether changes in media literacy and romantic-script internalization precede changes in SIRBS scores and related relationship beliefs.

Second, all variables were assessed using self-report questionnaires administered in the same survey (Podsakoff et al., 2003). Although the study included nomological, criterion-related, and incremental analyses, several external variables were developed for the present research context rather than drawn from well-established instruments. The original survey did not include established measures of romantic beliefs, general materialism, acceptance of controlling behavior, or egalitarian relationship attitudes; direct tests of convergence and incremental distinctiveness relative to these measures could therefore not be conducted. The observed associations should consequently be interpreted as preliminary evidence of theoretically coherent placement within a nomological network rather than as comprehensive external validation. Future research should test the SIRBS against established measures of relationship satisfaction, gender-role attitudes, acceptance of controlling behaviors, romantic beliefs, materialism, media literacy, psychological wellbeing, and anxiety or depressive symptoms. Multi-informant data, partner reports, behavioral indicators, longitudinal designs, and experimental or intervention-based studies would provide stronger evidence for external validity and practical utility.

Third, the sample consisted of Chinese college students, which may limit generalizability to other age groups, educational backgrounds, and cultural contexts. Because intimate relationship beliefs are shaped by cultural norms, socioeconomic conditions, gender systems, and media environments, future studies should validate the SIRBS in more diverse populations and examine its cross-cultural measurement invariance.

Fourth, the study focused primarily on romantic micro-dramas and short-form romantic media. Other digital contexts, including dating applications, reality dating shows, online fiction, livestreaming, influencer content, and algorithmically curated social media feeds, may convey different relationship scripts. Future research should extend the SIRBS framework to these platforms to better understand how varied digital media ecologies are associated with young adults' relationship beliefs.

Finally, although the present study provided evidence for internal consistency, factorial validity, factor-level convergence and discrimination, and measurement invariance, further psychometric work remains necessary (Boateng et al., 2018; DeVellis and Thorpe, 2022; Stefana et al., 2025). Content review was conducted by two educational psychology experts and cognitive debriefing involved 28 students; future studies should broaden this process by incorporating larger and more multidisciplinary panels that include relationship educators, counselors, youth workers, and young adults themselves. Test-retest reliability should be examined to establish temporal stability, and intervention studies should assess sensitivity to change. Future research should also determine whether the three SIRBS dimensions predict later relationship quality, conflict management, boundary recognition, autonomy, and egalitarian partnership outcomes.

## 5 Conclusions

This study provides initial evidence for the SIRBS as a concise multidimensional measure of sustainable intimate relationship beliefs among Chinese college students. The correlated three-factor structure distinguishes non-materialistic, low-control, and egalitarian-growth belief domains and provides a domain-sensitive tool for research on relationship beliefs, romantic scripts, media literacy, and digitally mediated youth contexts.

The nomological findings further showed that stronger SIRBS scores were associated with higher media literacy and lower unsustainable romantic-script internalization. The mediation results suggested that script internalization may partly account for the associations between digital romantic media-related experiences and SIRBS scores, whereas media literacy appeared to function more clearly as an upstream interpretive resource than as a moderator of the script-belief association.

Overall, the SIRBS provides a practical multidimensional measure for future research, education, and intervention evaluation. The three subscale scores should be prioritized for substantive interpretation; a total score may be reported as a secondary broad composite when its multidimensional basis is made explicit. Future studies should examine temporal stability, sensitivity to intervention, cross-cultural measurement invariance, and convergence with established measures of romantic beliefs, materialism, controlling-behavior acceptance, gender-role attitudes, relationship quality, psychological wellbeing, and later relational outcomes.

## Data Availability

The data supporting the findings of this study are available from the corresponding author upon reasonable request. The data are not publicly available due to privacy and ethical considerations concerning sensitive relationship- and marriage-related information.
