# Supplementary material for: Sustainable intimate relationship beliefs in digital romantic media contexts: development and psychometric validation of the SIRBS among Chinese college students
Source: Front Psychol. 2026 Oct 28;17:1929638. doi: 10.3389/fpsyg.2026.1929638 (PMC13752086; doi:10.3389/fpsyg.2026.1929638)
Supplement: Supplementary file 1 [file Supplementary_file_1.docx]

**Supplementary Materials**

**Table S1. Full Chinese items and English translations of the 12-item SIRBS.**

| **Item** | **Chinese wording** | **English translation** | **Dimension** | **Original coding** | **Scoring in SIRBS** |
| --- | --- | --- | --- | --- | --- |
| NM1 | 我希望未来伴侣有较强的经济实力 | I hope that my future partner will have strong financial resources. | NM | 1–5 | Reverse-coded |
| NM2 | 伴侣的收入和家庭条件会显著影响我的选择 | A partner’s income and family background would substantially influence my choice. | NM | 1–5 | Reverse-coded |
| NM3 | 稳定且优越的物质条件是亲密关系安全感的重要来源 | Stable and superior material conditions are an important source of security in intimate relationships. | NM | 1–5 | Reverse-coded |
| NM4 | 我认为高质量恋爱需要一定的消费水平 | I believe that a high-quality romantic relationship requires a certain level of consumption. | NM | 1–5 | Reverse-coded |
| LC1 | 恋爱中适度的占有欲说明对方在乎我 | Moderate possessiveness in a romantic relationship means that the partner cares about me. | LC | 1–5 | Reverse-coded |
| LC2 | 如果对方足够爱我，强势一点也是可以接受的 | If the partner loves me enough, being somewhat forceful is acceptable. | LC | 1–5 | Reverse-coded |
| LC3 | 伴侣干预我的一些选择有时可能是出于保护 | A partner’s interference with some of my choices may sometimes be protective. | LC | 1–5 | Reverse-coded |
| LC4 | 误会和拉扯有时会让感情更深 | Misunderstandings and emotional push-pull may sometimes deepen a relationship. | LC | 1–5 | Reverse-coded |
| EG1 | 健康恋爱应该建立在平等和尊重上 | A healthy romantic relationship should be based on equality and respect. | EG | 1–5 | Forward-coded |
| EG2 | 双方都应该保留独立空间和个人选择 | Both partners should maintain personal space and individual choices. | EG | 1–5 | Forward-coded |
| EG3 | 伴侣之间应通过沟通解决矛盾 | Partners should resolve conflicts through communication. | EG | 1–5 | Forward-coded |
| EG4 | 我更欣赏共同成长而不是单方面依赖的关系 | I value relationships based on mutual growth rather than one-sided dependence. | EG | 1–5 | Forward-coded |

*Note.* SIRBS = Sustainable Intimate Relationship Beliefs Scale; NM = Non-materialistic Relationship Beliefs; LC = Low Control Tolerance; EG = Egalitarian Growth Beliefs. Chinese items were administered to participants, and English translations are provided for reporting purposes. All items were rated from 1 = strongly disagree to 5 = strongly agree. NM and LC items were reverse-scored when computing SIRBS scores.

**Table S2. External validation measures used in the study.**

| **Construct** | **No. of items** | **Item content domain** | **Scoring** | **Cronbach’s α** |
| --- | --- | --- | --- | --- |
| Micro-drama exposure | 8 | Frequency or intensity of exposure to romantic micro-drama / short-form romantic media content | Mean score; higher scores indicate greater exposure | 0.766 |
| Narrative transportation | 6 | Emotional and cognitive immersion in romantic media narratives | Mean score; higher scores indicate stronger transportation | 0.903 |
| Character involvement | 6 | Identification with or emotional involvement in romantic media characters | Mean score; higher scores indicate stronger character involvement | 0.835 |
| Romantic reality gap | 6 | Perceived discrepancy or disappointment when comparing real or anticipated intimate relationships with idealized romantic narratives | Mean score; higher scores indicate a stronger reality gap | 0.915 |
| High-resource partner script | 4 | Endorsement of high-resource or materially advantaged partner expectations | Mean score; higher scores indicate stronger endorsement | 0.796 |
| Fated love script | 4 | Endorsement of destiny-oriented or fated-love romantic beliefs | Mean score; higher scores indicate stronger endorsement | 0.782 |
| Conflict compensation script | 4 | Endorsement of dramatic conflict repair or compensation scripts in romantic relationships | Mean score; higher scores indicate stronger endorsement | 0.792 |
| Reduced or reversed female agency script | 4 | Endorsement of scripts implying reduced or reversed female agency in romantic relationships | Reverse-scored mean; higher scores indicate stronger unsustainable agency-related script internalization | 0.802 |
| Unsustainable romantic-script internalization | 16 | Composite of high-resource partner script, fated love script, conflict compensation script, and reduced/reversed female agency script | Mean of the 16 scored items; higher scores indicate stronger unsustainable script internalization | 0.828 |
| Media literacy | 6 | Critical evaluation of romantic media content, including exaggeration, commercialization, algorithmic recommendation, and unrealistic relationship scripts | Mean score; higher scores indicate stronger media literacy | 0.892 |
| Marriage intention | 4 | Willingness or likelihood to consider marriage in the future | Mean score; higher scores indicate stronger marriage intention | 0.8 |
| Marriage anxiety | 4 | Concerns about marriage, including freedom, economic pressure, unequal responsibilities, and discrepancy between idealized love and marital reality | Mean score; higher scores indicate stronger marriage anxiety | 0.875 |

*Note.* All external measures were self-report scales rated on five-point Likert-type response formats. Scale scores were computed as item means, with higher scores indicating higher levels of the corresponding construct. Cronbach’s alpha is reported as the internal consistency estimate. The reduced or reversed female agency script items were reverse-scored before computing the corresponding subscale and the unsustainable romantic-script internalization composite. These measures were used for context-specific nomological and criterion-related analyses; several were developed for the present research context and should not be interpreted as established external convergent measures of the SIRBS.

**Table S2a. Item development and content-review process.**

| **Stage** | **Procedure** | **Evaluation focus** | **Outcome** |
| --- | --- | --- | --- |
| Conceptual specification | The construct was defined as a multidimensional orientation toward non-materialistic, low-control, egalitarian, mutually respectful, and growth-oriented intimate relationships. | Coverage of relational well-being, autonomy and boundary recognition, gender equality and egalitarian partnership, and critical media literacy regarding romantic media. | A four-domain framework guided candidate-item drafting. |
| Candidate-item drafting | Belief statements were written for use with Chinese college students and without requiring current relationship experience. | Conceptual coverage, comprehensibility, cultural relevance, and nonredundancy. | A preliminary item pool was prepared for expert review. |
| Expert review | Two experts in educational psychology reviewed the candidate items. | Conceptual relevance, clarity, cultural appropriateness, redundancy, suitability for Chinese college students, and alignment with the intended dimension. | Items were revised or removed when they showed conceptual overlap, ambiguous wording, or weak dimensional alignment. |
| Cognitive debriefing | Twenty-eight college students reviewed the items and response format. | Comprehensibility, consistency of interpretation, and correspondence with the intended meaning. | Wording was refined where necessary, and item interpretation was checked before administration. |
| Scale finalization | The reviewed items were organized into the three intended dimensions. | Balanced coverage of Non-materialistic Relationship Beliefs, Low Control Tolerance, and Egalitarian Growth Beliefs. | The final SIRBS contained 12 items, with four items per dimension. |

*Note.* SIRBS = Sustainable Intimate Relationship Beliefs Scale.

**Table S3a. Floor/ceiling effects and corrected item-total correlations.**

| **Item** | **Dimension** | **Floor %** | **Ceiling %** | **Corrected item-total r** | **α if item deleted** |
| --- | --- | --- | --- | --- | --- |
| NM1 | NM | 0.05 | 0.45 | .769 | .890 |
| NM2 | NM | 0.15 | 0.35 | .777 | .888 |
| NM3 | NM | 0.05 | 0.50 | .812 | .875 |
| NM4 | NM | 0.20 | 0.30 | .816 | .874 |
| LC1 | LC | 0.20 | 0.75 | .619 | .758 |
| LC2 | LC | 0.15 | 0.75 | .630 | .752 |
| LC3 | LC | 0.00 | 0.70 | .612 | .761 |
| LC4 | LC | 0.15 | 0.65 | .622 | .756 |
| EG1 | EG | 0.30 | 0.30 | .618 | .764 |
| EG2 | EG | 0.25 | 0.35 | .616 | .765 |
| EG3 | EG | 0.45 | 0.55 | .644 | .751 |
| EG4 | EG | 0.30 | 0.45 | .623 | .762 |

*Note.* NM = Non-materialistic Relationship Beliefs; LC = Low Control Tolerance; EG = Egalitarian Growth Beliefs. Floor and ceiling effects refer to the percentage of responses at the minimum and maximum response categories, respectively. Corrected item-total correlations and alpha-if-item-deleted values were computed within each four-item dimension.

**Table S3b-1. Response category distributions for the SIRBS items**

| **Item** | **1 n (%)** | **2 n (%)** | **3 n (%)** | **4 n (%)** | **5 n (%)** |
| --- | --- | --- | --- | --- | --- |
| **NM1** | 1 (0.0) | 272 (13.5) | 1266 (63.0) | 460 (22.9) | 9 (0.4) |
| **NM2** | 3 (0.1) | 259 (12.9) | 1293 (64.4) | 446 (22.2) | 7 (0.3) |
| **NM3** | 1 (0.0) | 269 (13.4) | 1267 (63.1) | 461 (23.0) | 10 (0.5) |
| **NM4** | 4 (0.2) | 275 (13.7) | 1247 (62.1) | 476 (23.7) | 6 (0.3) |
| **LC1** | 4 (0.2) | 189 (9.4) | 1240 (61.8) | 560 (27.9) | 15 (0.7) |
| **LC2** | 3 (0.1) | 182 (9.1) | 1240 (61.8) | 568 (28.3) | 15 (0.7) |
| **LC3** | 0 (0.0) | 163 (8.1) | 1257 (62.6) | 574 (28.6) | 14 (0.7) |
| **LC4** | 3 (0.1) | 160 (8.0) | 1263 (62.9) | 569 (28.3) | 13 (0.6) |
| **EG1** | 6 (0.3) | 312 (15.5) | 1262 (62.8) | 422 (21.0) | 6 (0.3) |
| **EG2** | 5 (0.2) | 302 (15.0) | 1250 (62.3) | 444 (22.1) | 7 (0.3) |
| **EG3** | 9 (0.4) | 301 (15.0) | 1230 (61.3) | 457 (22.8) | 11 (0.5) |
| **EG4** | 6 (0.3) | 299 (14.9) | 1292 (64.3) | 402 (20.0) | 9 (0.4) |

*Note.* N = 2008. Values are n (%). The response scale ranged from 1 = strongly disagree to 5 = strongly agree. NM and LC items were reverse-scored before analysis; EG items were forward-scored.

**Table S3b-2. Descriptive statistics and distribution diagnostics for the SIRBS items**

| **Item** | **M** | **SD** | **Skewness** | **Kurtosis** | **Floor (%)** | **Ceiling (%)** | **Midpoint (%)** |
| --- | --- | --- | --- | --- | --- | --- | --- |
| **NM1** | 3.102 | 0.612 | 0.045 | -0.026 | 0.0 | 0.4 | 63.0 |
| **NM2** | 3.097 | 0.601 | 0.013 | 0.126 | 0.1 | 0.3 | 64.4 |
| **NM3** | 3.105 | 0.612 | 0.056 | -0.001 | 0.0 | 0.5 | 63.1 |
| **NM4** | 3.102 | 0.619 | -0.044 | -0.053 | 0.2 | 0.3 | 62.1 |
| **LC1** | 3.196 | 0.611 | 0.010 | 0.134 | 0.2 | 0.7 | 61.8 |
| **LC2** | 3.204 | 0.607 | 0.028 | 0.090 | 0.1 | 0.7 | 61.8 |
| **LC3** | 3.219 | 0.589 | 0.109 | -0.038 | 0.0 | 0.7 | 62.6 |
| **LC4** | 3.214 | 0.591 | 0.048 | 0.147 | 0.1 | 0.6 | 62.9 |
| **EG1** | 3.055 | 0.622 | -0.037 | 0.051 | 0.3 | 0.3 | 62.8 |
| **EG2** | 3.073 | 0.625 | -0.028 | -0.002 | 0.2 | 0.3 | 62.3 |
| **EG3** | 3.080 | 0.641 | -0.050 | 0.107 | 0.4 | 0.5 | 61.3 |
| **EG4** | 3.054 | 0.613 | 0.008 | 0.261 | 0.3 | 0.4 | 64.3 |

*Note.* N = 2008. Floor refers to the percentage of responses in category 1; ceiling refers to category 5; midpoint refers to category 3. NM and LC items were reverse-scored before analysis; EG items were forward-scored.

**Table S3c. Data screening and quality-control summary**

| **Step** | **Criterion applied** | **Removed at step** | **Retained after step** | **Note** |
| --- | --- | --- | --- | --- |
| Initial submitted responses | All questionnaire submissions exported from the survey platform | 0 | 2152 |  |
| Eligibility: age | Age 18 or above | 1 | 2151 | One submitted response was excluded because the participant was younger than 18 years. |
| Eligibility: student status | Currently enrolled in a higher education institution | 0 | 2151 | All retained submitted cases met the student-status criterion. |
| SIRBS item completeness | Complete responses on all 12 SIRBS items | 0 | 2151 | No SIRBS item missingness was present among retained submitted cases. |
| Completion-time screen | Completion time of at least 120 seconds | 64 | 2087 | Responses shorter than 120 seconds were excluded before attention-check screening. |
| Attention check | Correct response to the instructed-response item: choose “比较同意” | 79 | 2008 | The correct numeric response was 4. |
| Final analytic sample | Eligible, complete SIRBS responses with valid completion time and a passed attention check |  | 2008 | This sample was used for the main psychometric analyses. |

*Note.* The attention-check item instructed participants to select '比较同意', coded as 4. The final analytic sample consisted of eligible participants with complete SIRBS responses who passed the attention check.

**Table S3d. Completion-time diagnostics**

| **Sample** | **N** | **Completion-time criterion or summary** | **Status** | **Note** |
| --- | --- | --- | --- | --- |
| Submitted responses after age eligibility screening | 2151 | Threshold = 120 seconds | Before completion-time screening | One underage submitted response had already been excluded. |
| Excluded by completion-time screen | 64 | < 120 seconds | Excluded | Removed before attention-check screening. |
| Retained after completion-time screen | 2087 | ≥ 120 seconds | Retained | Screened further for attention-check performance. |
| Final analytic sample | 2008 | M = 815.54, SD = 50.39; median = 817; range = 365–1063 | Retained | Used for the main analyses after attention-check screening. |

*Note.* Completion time is reported in seconds as recorded by the survey platform. The 120-second threshold was applied after age eligibility screening and before attention-check screening.

**Table S3e. Reliability estimates for the final analytic sample.**

| **Sample** | **N** | **Total SIRBS alpha** | **NM alpha** | **LC alpha** | **EG alpha** |
| --- | --- | --- | --- | --- | --- |
| Final analytic sample after age, completion-time, and attention-check exclusions | 2008 | 0.8452 | 0.9087 | 0.8057 | 0.8089 |

*Note.* Cronbach's alpha values were computed using SIRBS-scored items*.*

**Table S3f. Pearson inter-item correlation matrix for the SIRBS**

| **Item** | **NM1** | **NM2** | **NM3** | **NM4** | **LC1** | **LC2** | **LC3** | **LC4** | **EG1** | **EG2** | **EG3** | **EG4** |
| --- | --- | --- | --- | --- | --- | --- | --- | --- | --- | --- | --- | --- |
| NM1 | 1 | 0.661 | 0.71 | 0.717 | 0.26 | 0.274 | 0.282 | 0.251 | 0.167 | 0.142 | 0.177 | 0.158 |
| NM2 | 0.661 | 1 | 0.721 | 0.724 | 0.286 | 0.306 | 0.282 | 0.272 | 0.172 | 0.15 | 0.184 | 0.186 |
| NM3 | 0.71 | 0.721 | 1 | 0.747 | 0.267 | 0.306 | 0.289 | 0.278 | 0.181 | 0.153 | 0.169 | 0.176 |
| NM4 | 0.717 | 0.724 | 0.747 | 1 | 0.259 | 0.291 | 0.287 | 0.267 | 0.165 | 0.147 | 0.17 | 0.166 |
| LC1 | 0.26 | 0.286 | 0.267 | 0.259 | 1 | 0.515 | 0.497 | 0.511 | 0.169 | 0.19 | 0.212 | 0.183 |
| LC2 | 0.274 | 0.306 | 0.306 | 0.291 | 0.515 | 1 | 0.512 | 0.519 | 0.225 | 0.196 | 0.216 | 0.215 |
| LC3 | 0.282 | 0.282 | 0.289 | 0.287 | 0.497 | 0.512 | 1 | 0.501 | 0.189 | 0.19 | 0.198 | 0.159 |
| LC4 | 0.251 | 0.272 | 0.278 | 0.267 | 0.511 | 0.519 | 0.501 | 1 | 0.212 | 0.181 | 0.207 | 0.202 |
| EG1 | 0.167 | 0.172 | 0.181 | 0.165 | 0.169 | 0.225 | 0.189 | 0.212 | 1 | 0.493 | 0.529 | 0.507 |
| EG2 | 0.142 | 0.15 | 0.153 | 0.147 | 0.19 | 0.196 | 0.19 | 0.181 | 0.493 | 1 | 0.525 | 0.506 |
| EG3 | 0.177 | 0.184 | 0.169 | 0.17 | 0.212 | 0.216 | 0.198 | 0.207 | 0.529 | 0.525 | 1 | 0.525 |
| EG4 | 0.158 | 0.186 | 0.176 | 0.166 | 0.183 | 0.215 | 0.159 | 0.202 | 0.507 | 0.506 | 0.525 | 1 |

*Note.* Correlations were computed using SIRBS-scored items in the final analytic sample.

**Table S3g. Polychoric inter-item correlation matrix for the SIRBS**

| **Item** | **NM1** | **NM2** | **NM3** | **NM4** | **LC1** | **LC2** | **LC3** | **LC4** | **EG1** | **EG2** | **EG3** | **EG4** |
| --- | --- | --- | --- | --- | --- | --- | --- | --- | --- | --- | --- | --- |
| NM1 | 1 | 0.83 | 0.873 | 0.879 | 0.333 | 0.35 | 0.362 | 0.326 | 0.214 | 0.181 | 0.225 | 0.202 |
| NM2 | 0.83 | 1 | 0.884 | 0.885 | 0.37 | 0.393 | 0.367 | 0.357 | 0.223 | 0.192 | 0.235 | 0.24 |
| NM3 | 0.873 | 0.884 | 1 | 0.903 | 0.343 | 0.393 | 0.373 | 0.363 | 0.233 | 0.196 | 0.214 | 0.226 |
| NM4 | 0.879 | 0.885 | 0.903 | 1 | 0.331 | 0.372 | 0.37 | 0.347 | 0.212 | 0.186 | 0.217 | 0.213 |
| LC1 | 0.333 | 0.37 | 0.343 | 0.331 | 1 | 0.661 | 0.642 | 0.659 | 0.217 | 0.242 | 0.271 | 0.236 |
| LC2 | 0.35 | 0.393 | 0.393 | 0.372 | 0.661 | 1 | 0.67 | 0.673 | 0.29 | 0.251 | 0.275 | 0.275 |
| LC3 | 0.362 | 0.367 | 0.373 | 0.37 | 0.642 | 0.67 | 1 | 0.657 | 0.246 | 0.244 | 0.254 | 0.206 |
| LC4 | 0.326 | 0.357 | 0.363 | 0.347 | 0.659 | 0.673 | 0.657 | 1 | 0.274 | 0.234 | 0.265 | 0.26 |
| EG1 | 0.214 | 0.223 | 0.233 | 0.212 | 0.217 | 0.29 | 0.246 | 0.274 | 1 | 0.626 | 0.667 | 0.642 |
| EG2 | 0.181 | 0.192 | 0.196 | 0.186 | 0.242 | 0.251 | 0.244 | 0.234 | 0.626 | 1 | 0.657 | 0.644 |
| EG3 | 0.225 | 0.235 | 0.214 | 0.217 | 0.271 | 0.275 | 0.254 | 0.265 | 0.667 | 0.657 | 1 | 0.666 |
| EG4 | 0.202 | 0.24 | 0.226 | 0.213 | 0.236 | 0.275 | 0.206 | 0.26 | 0.642 | 0.644 | 0.666 | 1 |

*Note.* Polychoric correlations were estimated pairwise from the five-category SIRBS-scored items. They are intended as supplementary ordinal-association diagnostics.

**Table S4. Exploratory factor analysis pattern matrix and communalities in the EFA subsample (n = 1004).**

| **Item** | **NM** | **LC** | **EG** | **Communality (h²)** |
| --- | --- | --- | --- | --- |
| NM1 | .814 | -.008 | .016 | .665 |
| NM2 | .824 | .050 | -.013 | .714 |
| NM3 | .875 | -.002 | .016 | .773 |
| NM4 | .887 | -.028 | -.016 | .757 |
| LC1 | .001 | .716 | .025 | .526 |
| LC2 | .016 | .707 | .005 | .513 |
| LC3 | .022 | .667 | .017 | .468 |
| LC4 | -.022 | .752 | -.032 | .535 |
| EG1 | -.011 | .034 | .709 | .516 |
| EG2 | -.023 | -.002 | .745 | .545 |
| EG3 | .020 | -.037 | .752 | .557 |
| EG4 | .014 | .016 | .726 | .541 |

*Note.* EFA = exploratory factor analysis; NM = Non-materialistic Relationship Beliefs; LC = Low Control Tolerance; EG = Egalitarian Growth Beliefs; h² = communality. Principal axis factoring with oblimin rotation was used. KMO = .876; Bartlett’s test of sphericity: χ²(66) = 5638.542, p < .001.

**Table S5. Latent/scale correlations and HTMT ratios for discriminant validity.**

| **Construct** | **NM** | **LC** | **EG** |
| --- | --- | --- | --- |
| NM | .900 | .396 | .236 |
| LC | .462 | .742 | .310 |
| EG | .275 | .384 | .721 |

*Note.* NM = Non-materialistic Relationship Beliefs; LC = Low Control Tolerance; EG = Egalitarian Growth Beliefs; AVE = average variance extracted; HTMT = heterotrait-monotrait ratio. Diagonal values are the square roots of AVE. Upper-triangle values are correlations among the three SIRBS dimensions. Lower-triangle values are HTMT ratios.

**Table S6. Group comparison diagnostics after measurement invariance testing.**

| **Grouping variable** | **Construct** | **Reference group** | **Comparison group** | **Mref** | **Mcomp** | **ΔM** | **SE** | **t** | **p** | **Cohen’s d** |
| --- | --- | --- | --- | --- | --- | --- | --- | --- | --- | --- |
| Gender | NM | Male | Female | 3.098 | 3.103 | .005 | .024 | -.206 | .837 | .009 |
| Gender | LC | Male | Female | 3.192 | 3.219 | .027 | .021 | -1.263 | .207 | .056 |
| Gender | EG | Male | Female | 3.065 | 3.065 | .001 | .023 | -.030 | .976 | .001 |
| Gender | SIRBS | Male | Female | 3.118 | 3.129 | .011 | .017 | -.647 | .518 | .029 |
| Relationship experience | NM | No experience | Experience | 3.098 | 3.102 | .004 | .029 | -.129 | .898 | .007 |
| Relationship experience | LC | No experience | Experience | 3.208 | 3.208 | -.000 | .025 | .014 | .988 | -.001 |
| Relationship experience | EG | No experience | Experience | 3.069 | 3.064 | -.004 | .027 | .160 | .873 | -.009 |
| Relationship experience | SIRBS | No experience | Experience | 3.125 | 3.125 | -.000 | .020 | .018 | .985 | -.001 |

*Note.* NM = Non-materialistic Relationship Beliefs; LC = Low Control Tolerance; EG = Egalitarian Growth Beliefs; SIRBS = Sustainable Intimate Relationship Beliefs Scale; Mref = mean of the reference group; Mcomp = mean of the comparison group; ΔM = comparison group mean minus reference group mean; SE = standard error. Comparisons were based on observed scale scores after scalar invariance was supported.

**Table S7a. Incremental validity models predicting marriage intention and marriage anxiety.**

| **Outcome** | **Model** | **Predictors added** | **R²** | **Adjusted R²** | **ΔR²** | **F change** | **p change** |
| --- | --- | --- | --- | --- | --- | --- | --- |
| Marriage Intention | Step 1 | Demographics | .003 | .002 | — | — | — |
| Marriage Intention | Step 2 | Media/script variables | .010 | .005 | .007 | 2.292 | .033 |
| Marriage Intention | Step 3 | SIRBS | .038 | .033 | .028 | 58.349 | < .001 |
| Marriage Anxiety | Step 1 | Demographics | .002 | .001 | — | — | — |
| Marriage Anxiety | Step 2 | Media/script variables | .151 | .147 | .148 | 58.138 | < .001 |
| Marriage Anxiety | Step 3 | SIRBS | .152 | .148 | .002 | 3.692 | .055 |

*Note.* R² = coefficient of determination; ΔR² = change in R². Step 1 included gender, age group, and romantic relationship experience. Step 2 added micro-drama exposure, narrative transportation, character involvement, romantic reality gap, media literacy, and unsustainable romantic-script internalization. Step 3 added the total SIRBS score.

**Table S7b. Final-step coefficients for the incremental contribution of SIRBS.**

| **Outcome** | **Predictor** | **B** | **SE** | **t** | **p** |
| --- | --- | --- | --- | --- | --- |
| Marriage Intention | SIRBS | .306 | .040 | 7.639 | < .001 |
| Marriage Anxiety | SIRBS | -.079 | .041 | -1.922 | .055 |

*Note.* SIRBS = Sustainable Intimate Relationship Beliefs Scale; B = unstandardized coefficient; SE = standard error. Coefficients are reported from the final regression model after demographic covariates and media/script variables were controlled.

**Table S8. Exploratory moderation analysis: media literacy as a moderator of the script-internalization–SIRBS association.**

| **Predictor** | **B** | **SE** | **t** | **p** |
| --- | --- | --- | --- | --- |
| Script | -.411 | .018 | -22.876 | < .001 |
| Media Literacy | .258 | .017 | 14.950 | < .001 |
| Exposure | -.031 | .018 | -1.769 | .077 |
| Transportation | -.088 | .021 | -4.214 | < .001 |
| Character | -.062 | .020 | -3.102 | .002 |
| Reality Gap | -.135 | .022 | -6.242 | < .001 |
| Script x Media Literacy | .013 | .014 | 0.903 | .366 |

*Note.* SIRBS = Sustainable Intimate Relationship Beliefs Scale; B = unstandardized coefficient; SE = standard error. The dependent variable was the standardized SIRBS score. Continuous predictors were standardized before the interaction term was computed. Script = unsustainable romantic-script internalization; Media Literacy = media literacy; Exposure = micro-drama exposure; Transportation = narrative transportation; Character = character involvement; Reality Gap = romantic reality gap; Script x Media Literacy = interaction between unsustainable romantic-script internalization and media literacy.

**Table S9a. Common method bias diagnostics.**

| **Diagnostic** | **Index** | **Value** | **Interpretation** |
| --- | --- | --- | --- |
| Harman single-factor test | First unrotated factor variance | 21.9% | Below 40–50% diagnostic threshold |
| Single-factor CFA diagnostic | CFI | .457 | Poor fit |
| Single-factor CFA diagnostic | TLI | .438 | Poor fit |
| Single-factor CFA diagnostic | RMSEA | .095 | Poor fit |
| Single-factor CFA diagnostic | SRMR | .105 | Poor fit |

*Note.* CFA = confirmatory factor analysis; CFI = comparative fit index; TLI = Tucker–Lewis index; RMSEA = root mean square error of approximation; SRMR = standardized root mean square residual. The single-factor CFA diagnostic model included the SIRBS items and the external validation variables/items, excluding demographic variables and the attention-check item.

**Table S9b. Robustness check using 50 random split-half CFA replications.**

| **Fit index** | **Mean** | **SD** |
| --- | --- | --- |
| CFI | 0.992 | 0.004 |
| TLI | 0.989 | 0.006 |
| RMSEA | 0.034 | 0.008 |
| SRMR | 0.028 | 0.004 |

*Note.* CFA = confirmatory factor analysis; CFI = comparative fit index; TLI = Tucker–Lewis index; RMSEA = root mean square error of approximation; SRMR = standardized root mean square residual; SD = standard deviation. In each replication, the full sample was randomly split and the three-factor CFA model was re-estimated.
